# Supplementary material for: Ferroelectric nematic liquids with conics
Source: Nat Commun. 2023 Feb 10;14:748. doi: 10.1038/s41467-023-36326-1 (PMC9918734; doi:10.1038/s41467-023-36326-1)
Supplement: Supplementary file 1 — Supplementary Information [file 41467_2023_36326_MOESM1_ESM.pdf]

SUPPLEMENTARY INFORMATION

Ferroelectric nematic liquids with conics

Priyanka Kumari[1,2], Bijaya Basnet[1,2], Hao Wang[1], and Oleg D. Lavrentovich[1,2,3]*

Affiliations:

[1]*Advanced Materials and Liquid Crystal Institute, Kent State University, Kent, OH 44242, USA*

[2]*Materials Science Graduate Program, Kent State University, Kent, OH 44242, USA*

[3]*Department of Physics, Kent State University, Kent, OH 44242, USA*

Corresponding author:

*Author for correspondence: e-mail: olavrent@kent.edu, tel.: +1-330-672-4844.

Keywords: ferroelectric nematic liquid crystal, domain walls, conic sections

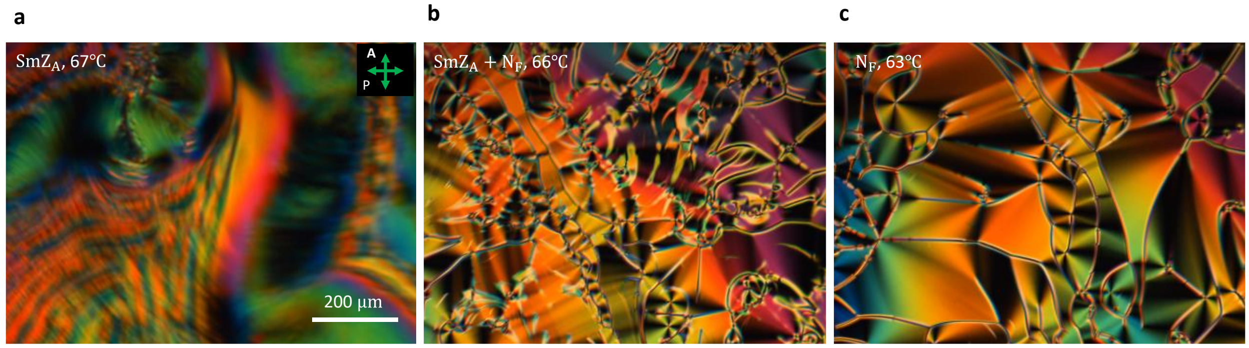

Supplementary Fig. 1 Textural changes during the SmZ$_A$ - N$_F$ phase transition. a SmZ$_A$ texture. **b** Coexistence of SmZ$_A$ and N$_F$. **c** N$_F$ polygonal texture recorded a few seconds after the transition completion. Note numerous +1 circular disclinations with four dark brushes emanating from their cores and the network of domain walls.

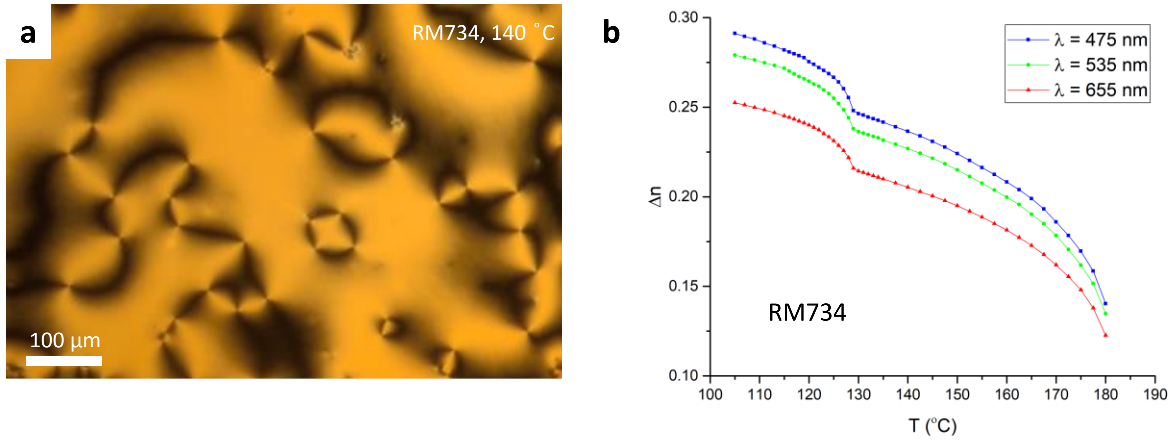

Supplementary Fig. 2 Schlieren texture of RM734 sandwiched between two glass plates; cell thickness $h = 1.7$ μm**. a** N phase with multiple +1/2 and -1/2 disclinations; each disclination core shows two extinction brushes. The disclination cores are isolated, which proves that the director is strictly parallel to the bounding plates. There is no preferred orientation in the plane of the cell. Figure 3 shows the same area of the sample in the N$_F$ phase; the optical retardance of the N$_F$ texture in Figure 3 is higher than the retardance of the N texture in part (**a**). **b** Temperature dependencies of RM734 birefringence measured by PolScope Microimager at wavelengths 475 nm, 535 nm, and 655 nm.

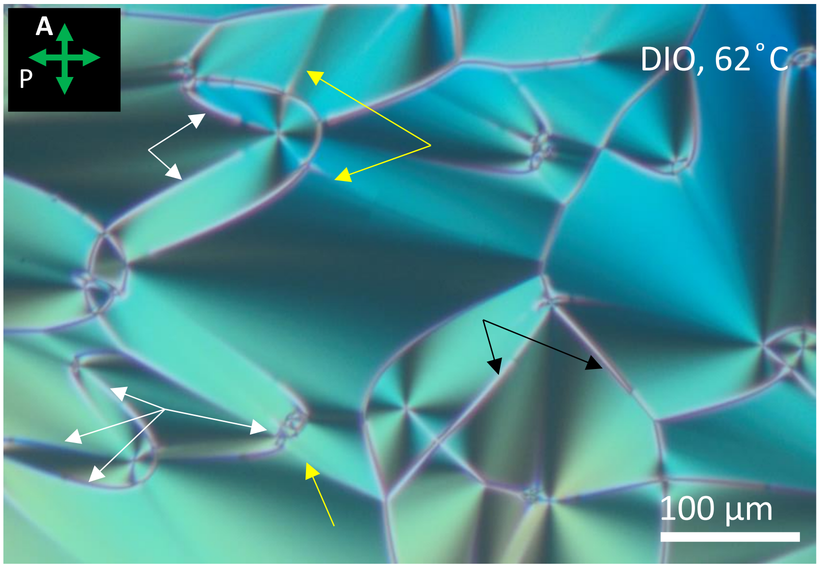

Supplementary Fig. 3 Texture of an N_F film of an average thickness $h \approx 5.9$ µm **after 10 min thermal equilibration.** Multiple +1 circular vortices and a network of conical DWs. White arrows point towards conics emanating from the cores of +1 disclinations; yellow arrows point towards conics that originate at the cores of -1/2 disclinations; black arrows show straight H-walls.

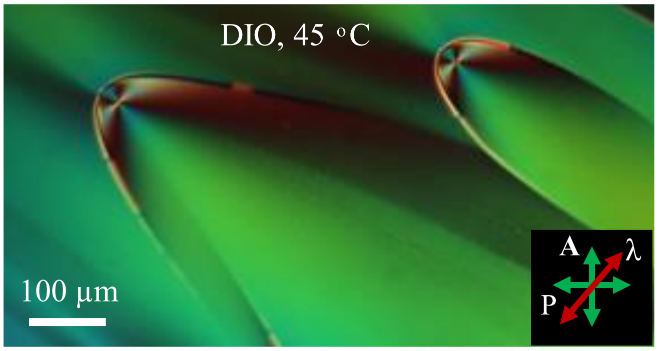

Supplementary Fig. 4 Texture of a DIO N_F film of an average thickness $h \approx 4.6$ µm**.** The texture is viewed between crossed polarizers and a 550 nm optical compensator with the slow axis along the bisectrix of the North-East and South-West quadrants.

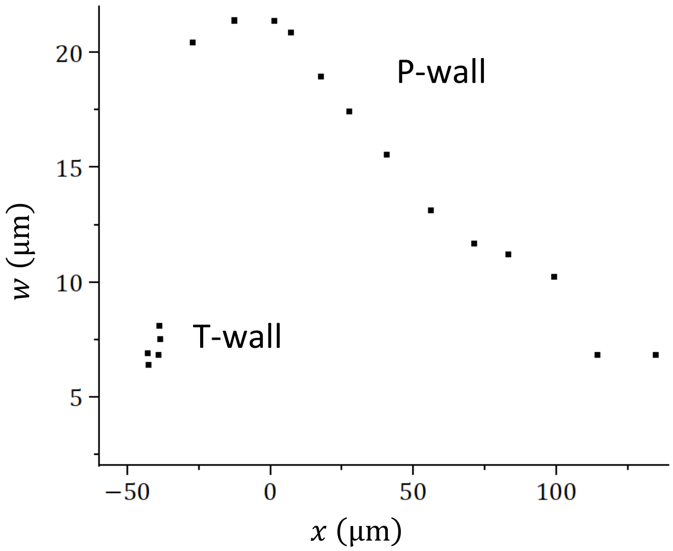

Supplementary Fig. 5 Thickness variation of the P- and T-walls in Fig.1g. The thickness of the P-wall increases as one moves towards the tip; the T-wall is narrow.

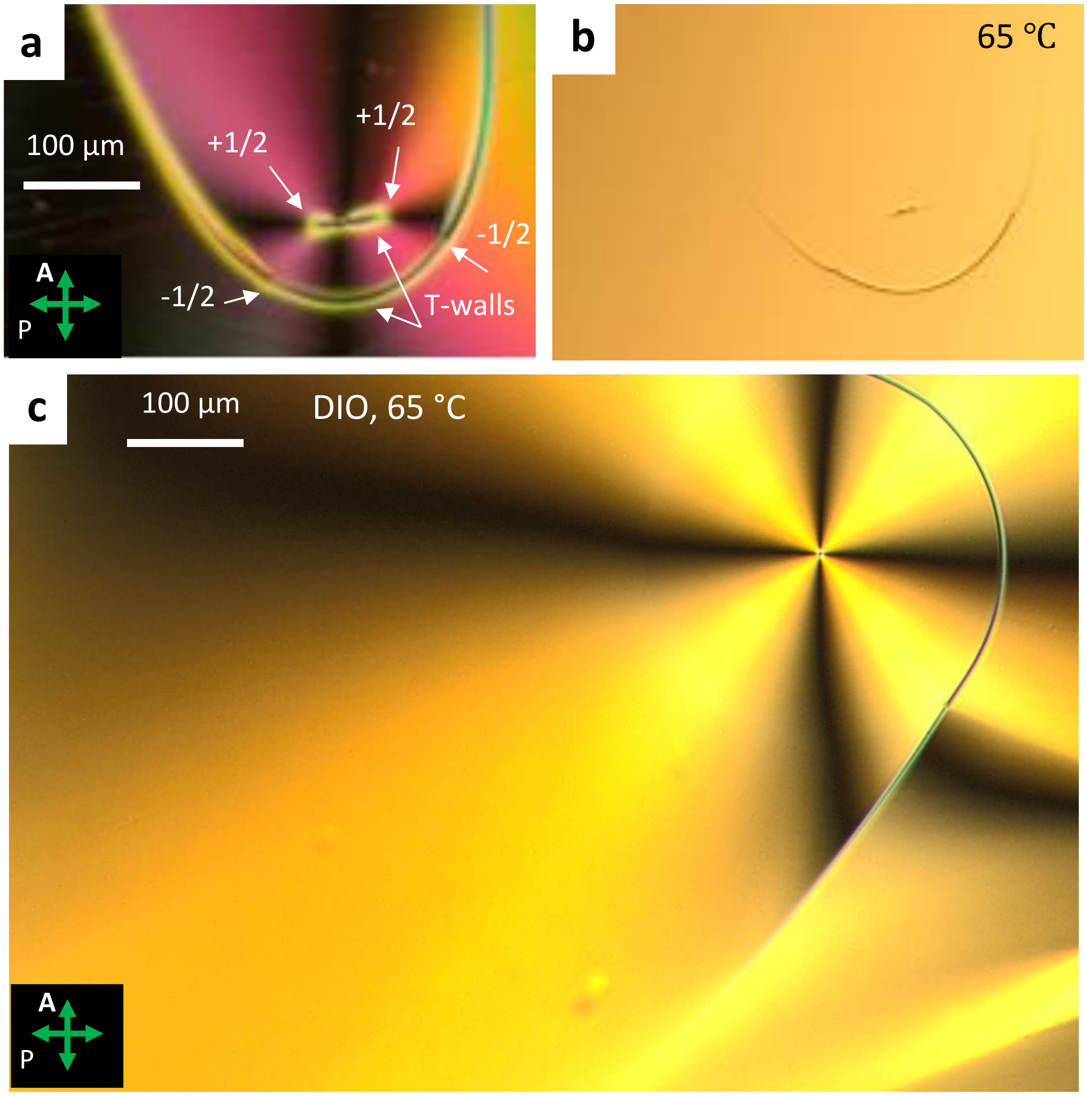

Supplementary Fig. 6 T-walls. a A pair of T-walls: One connects two +1/2 disclinations, another one connects two -1/2 disclinations. **b** The same as (**a**) but in unpolarized light; the optical contrast of the two T-walls is about the same which suggests a similar core structure. **c** A T-wall enclosing a circular vortex; it does not show full extinction in the region where it is parallel to the analyzer, which suggests a complex character of director distortions.

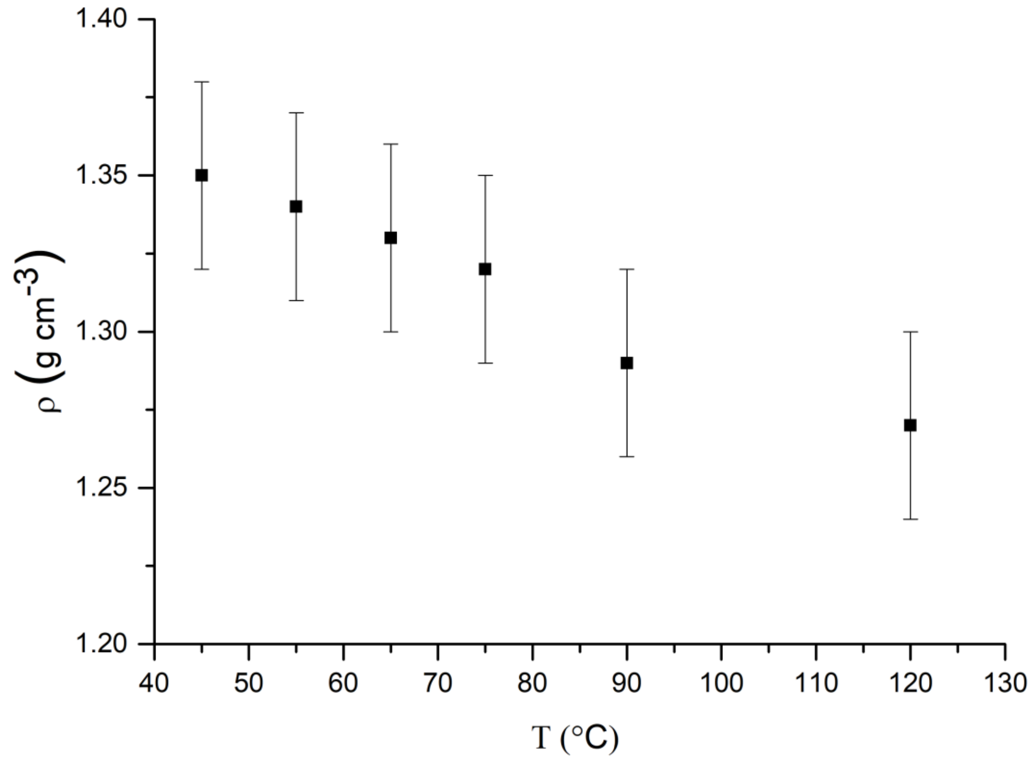

Supplementary Fig. 7 Temperature dependence of the DIO density. The error bars represent the instrumental error in measuring the cell thickness and the area occupied by the material.

The graph in Figure 4c was plotted using a Mathematica code

```
Plot[{2 ∗ ArcCot[Sqrt[x]], 2 ∗ ArcCot[1.5 ∗ Sqrt[((x ∗ (1.5 − 1) + 1)^2 − 1)/(1.5^2 − 1)]], 2 ∗
ArcCot[2 ∗ Sqrt[((x ∗ (2 − 1) + 1)^2 − 1)/(2^2 − 1)]], 2 ∗ ArcCot[3 ∗
Sqrt[((x ∗ (3 − 1) + 1)^2 − 1)/(3^2 − 1)]]}, {x, 0,3}, PlotRange−>
{{0,2.5}, {0,3.2}}, Ticks → {All, {0, Pi/4 , Pi/2 , 6 ∗ Pi/8 , Pi}}, AxesLabel → {x/f , "δ = π −
2θ"}, PlotStyle → {Black, Red, Blue, Orange}, Axes → True, AxesStyle → Black, PlotLabel →
None, LabelStyle → {12, GrayLevel[0]}, PlotLegends → Placed[{e_P" = 1", e_H" = 1.5", e_H" =
2", e_H" = 3"}, {0.7,0.7}]]
```
