# Supplementary material for: Ferroelectric nematic liquids with conics
Source: Nat Commun. 2023 Feb 10;14:748. doi: 10.1038/s41467-023-36326-1 (PMC9918734; doi:10.1038/s41467-023-36326-1)
Supplement: Supplementary file 3 — Description of Additional Supplementary Files [file 41467_2023_36326_MOESM3_ESM.docx]

**Description of Additional Supplementary Files**

**File Name: Supplementary Movie 1
Description:** DC electric field realigns the polarization field of DIO NF parallel to itself; the P-wall reorients its symmetry axis perpendicularly to the field.

**File Name: Supplementary Movie 2
Description:** Shear of a planar RM734 NF cell; DWs shift but do not split into separate lines.
